# Gut microbiome is associated with personality traits of free-ranging Tibetan macaques (*Macaca thibetana*)

**DOI:** 10.3389/fmicb.2024.1381372

**Published:** 2024-04-22

**Authors:** Mengyi Xia, Yingna Xia, Yu Sun, Jingjing Wang, Jiakai Lu, Xi Wang, Dongpo Xia, Xiaojuan Xu, Binghua Sun

**Affiliations:** ^1^School of Resources and Environmental Engineering, Anhui University, Hefei, China; ^2^International Collaborative Research Center for Huangshan Biodiversity and Tibetan Macaque Behavioral Ecology, Anhui University, Hefei, China; ^3^School of Life Sciences, Anhui University, Hefei, China; ^4^School of Biology and Food Engineering, Hefei Normal University, Hefei, China

**Keywords:** personality, gut microbiome, Tibetan macaque, free-ranging, diversity

## Abstract

Recent studies have emphasized that there is a strong link between the gut microbiome and the brain that affects social behavior and personality in animals. However, the interface between personality and the gut microbiome in wild primates remains poorly understood. Here, we used high-throughput sequencing and ethological methods in primate behavioral ecology to investigate the relationship between gut microbiome and personality in Tibetan macaques (*Macaca thibetana*). The behavioral assessment results indicated three personality dimensions including socialization, shyness, and anxiety. There was significant variation in alpha diversity only for shyness, with a significantly lower alpha diversity indices (including Shannon, Chao1, and PD) for bold individuals than for shy individuals. Using regression models to control for possible confounding factors, we found that the relative abundance of three genera, *Akkermansia*, *Dialister*, and *Asteroleplasma*, was significantly and positively correlated with the sociability scores in the macaques. In addition, Oscillospiraceae exhibited a positive correlation with scores for Shy Dimension. Furthermore, we found that the predicted functional genes for propionate and pyruvate, porphyrin and chlorophyll metabolic pathways related to animal behavior, were significant enriched in shyness group. We propose that the gut microbiome may play an important role in the formation of personality of Tibetan macaques.

## 1 Introduction

Behavioral differences among adults in the same population are usually consistent across time and context, and such behavioral differences, for example, behaviors related to aggression and friendliness, are often defined as personality traits of animals (Wolf and Weissing, 2012; Weiss et al., 2015). In a wide range of animal taxa, including humans, studies have shown that personality can influence behavioral changes that are critical to their survival (Altschul et al., 2018), reproduction (Rangassamy et al., 2015), distribution (Dinuzzo and Griffen, 2020), and ecological adaptations (Wolf and Weissing, 2012). Numerous previous studies have focused on the influence of various intrinsic and extrinsic factors on the process of personality shaping in animals, including genetics (Hancock et al., 2019), sex (Gartner and Powell, 2012), environment (Wei et al., 2017), and physiology (Peerboom and Wierenga, 2021). For example, changes in neurotransmitter and hormone levels are closely related to animal behavior, and influence the animal personality traits (Lynch and Hsiao, 2023). Given that gut microbes can produce a wide range of neuroactive chemicals and can also modulate the levels of neurotransmitters in the host (Silva et al., 2020; Lynch and Hsiao, 2023), the relationship between gut microbiome and animal personality traits has attracted attention in recent years.

In the last decade, there has been growing evidence that the gut microbiome plays an important role in animal neurological development (Sharon et al., 2016; Ali et al., 2019), physiological processes (Cryan et al., 2019) and behavioral occurrences (Davidson et al., 2018) in animals. For example, studies in humans and animals have shown that the gut microbiome can influence host social behaviors (Sgritta et al., 2019), stress responses (Savignac et al., 2014), anxiety and depressive symptoms (Taylor et al., 2020) through microbiome-gut-brain axis communication. In addition, imbalances in gut microbiome may result in neurotransmitter imbalances, inflammation, or enhanced activity of the hypothalamic- pituitary–adrenal axis (HPA); which regulates the stress response (Ortega et al., 2021; Chidambaram et al., 2022). Moreover, the gut microbiome can influence host behavior through chemical communication of their metabolites, such as short-chain fatty acids, with the nervous system (Liu et al., 2020). Additionally, it has been found that the composition of gut microbiome can be altered by direct recognition of stress hormones such as norepinephrine and epinephrine (Li et al., 2019; Barandouzi et al., 2022). Thus, there may be a strong link between gut microbiome and the personality of the animal.

Fecal microbial transplantation (FMT) offers valuable insights into the relationship between gut microbiome and personality. For example, it has been reported that sterile rat models transplanted with fecal microorganisms from shy and bold rats exhibit behavioral traits similar to those of their predecessors (Gan et al., 2022). Another study has shown that after colonizing the gut microbiome of people with anxiety and depressive symptoms, rodents exhibit human-like symptoms (Kelly et al., 2016). In recent years, a growing body of research has supported a strong relationship between gut microbiome and human personality. For example, a study in humans found that women’s and men’s anxiety levels were inversely related to *Bifidobacterium* and *Lactobacillus*, respectively (Taylor et al., 2020). Kim et al. (2018) found that Gammaproteobacteria and Proteobacteria were positively associated with neuroticism and conscientiousness when controlling for variables such as age, sex, BMI, and nutrient intake. Moreover, the composition and diversity of gut microbiome are related to the personality characteristics of human infants (Aatsinki et al., 2019), and adult individuals’ negative emotions are negative correlation with the alpha diversity of gut microbiome significantly (Kelsey et al., 2021). Previous studies in humans and laboratory animals have suggested that gut microbiome may be an important factor in the formation of animal personality, but this factor has not yet been fully revealed in a wider range of animal taxa.

Non-human primates (NHPs), a major group within the order of mammalia, have always been valuable model systems for the study of human behavior, physiology and health (Blaszczyk, 2020). Studies of wild populations such as common marmosets (*Callithrix jacchus*), white-faced capuchins (*Cebus capucinus*), and chimpanzees (*Pan troglodytes*) have demonstrated that NHPs exhibit a certain degree of individual behavioral variability, making them ideal subjects for exploring wild animal personality (Brandao et al., 2019; Rawlings et al., 2020; Slipogor et al., 2021). Previous studies have verified that individual differences are highly stable in adulthood in most rhesus macaques (Suomi et al., 1996). In particular, research conducted on Tibetan macaques (*Macaca thibetana*) has highlighted the diverse behaviors, frequent inter-individual interactions, intricate social relationships, and a subset of personality characteristics in this species (Pritchard et al., 2014). Therefore, the wild Tibetan macaques have the potential to offer critical insight into the interface between personality and gut microbiome that are missing from previous studies.

The social group of Tibetan macaques inhabiting the Mt. Huangshan in Anhui Province has been the subject of behavioral studies since 1986, providing a good opportunity to assess the link between the gut microbiome and personality in NHPs. In this study, we used high-throughput sequencing and research methods in primate behavioral ecology to investigate the relationship between gut microbiome and personality in Tibetan macaques. Our primary objectives were to (1) describe the composition of the fecal microbiome and personality traits of individual group members; (2) test whether personality traits is associated with the diversity, composition, and predicted functional metagenomes of the Tibetan macaque gut microbiome, taking into account age and sex, and (3) present these results in the context of what is known about the relationship between the gut microbiome of other mammals and animal behavior, and discuss the gut’s potential role in personality formation in Tibetan macaques.

## 2 Materials and methods

### 2.1 Samples collection and ethics statement

This study was conducted in the Mt. Huangshan National Reserve located in southern part of Anhui Province, China (30°29’N, 118°10′E), which is a highly seasonal ecosystem with an annual mean temperature of 15.3°C. The average elevation of the study area is 500 m above sea level, with a maximum elevation of 1,310 m. The study group named Yulinkeng 1 (YA1) has been habituated and monitored continuously since 1986. Due to the long history of the group being used for behavioral research, all individuals can be identified by specific physical characteristics. The ages of all individuals born in the group in the last 36 years are known, and the ages of all immigrants are estimated from information on individuals of known age in the group. During the period of our study, the group consisted of 61 individuals, including 27 adults (12 males and 15 females) and 34 juveniles (22 males and 12 females). Only 24 identified adults were included in this study, as the three newly migrated individuals often moved around the edges of the group, making it difficult to collect fecal samples from them. Information on all individuals involved in this study is presented in Supplementary Table S1.

We obtained a total of 94 fresh fecal samples (*x* = mean ± SD, *x* = 3.92 ± 0.40) from the 24 identified adult individuals (with females aged >5 years and males aged >7 years), including 15 females and 9 males. Fecal samples were collected between February 2023 and May 2023 from all age/sex classes. All fecal samples were collected, stored, and transported in the RNAlater (QIA-GEN, Valencia, CA, United States). The samples were frozen under −80°C at the field research base. Our samples were transported at ambient temperature, but were then stored at −80°C until DNA extraction began. This research was approved by the Institutional Animal Care and Use Committee of the Anhui Zoological Society (permit number BH20221203).

### 2.2 DNA extraction and sequencing

We extracted DNA from frozen fecal samples using the QIAamp^®^ Fast DNA Stool Mini Kit (Qiagen) and strictly followed the protocol specified by the manufacturer. Primers 338F (5′-ACTCCTACGGGAGGCAGCAGG-3′) and 806R (5′-GGACTACHVGGGTWTCTAAT-3′) were used to amplify the V3–V4 region of the 16S rRNA gene (Mori et al., 2014). PCR reaction mixtures contained 5–100 ng of DNA template, 1 × GoTaq Green master mix, 1 M MgCl_2_, and 5 pmol of each primer. The PCR reaction conditions included 3 min of pre-denaturation at 95°C, followed by 35 cycles of 95°C for 40 s, 52°C for 30 s, 72°C for 50 s, and a final extension at 72°C for 7 min. The PCR products underwent validation through agarose gel electrophoresis. After the individual quantification step, amplicons were pooled in equal amounts, and pair-end 2 × 300 bp sequencing was performed using the Illumina Miseq platform (San Diego, CA, United States) at Majorbio Bio-Pharm Technology Co., Ltd., Shanghai, China.

### 2.3 Personality assessment

To prevent any negative effects on the monkey colony, recording staff should maintain an appropriate distance from the colony when collecting behavioral data utilizing a digital voice recorder (model News my V03) and a DV camera (FDR-APX55, Sony Corporation, Tokyo, Japan). All the data were collected when the monkeys were in the natural forest, without the influence of human activity. Focal animal sampling was used to record randomly selected subjects for 15 min. We will select another individual randomly if the focal individual could not be followed or was lost from view during the sampling period. During the next 15 min sampling period, we will effort to locate and record the behavior of the lost individual. The data we collected did not include the chaotic events caused by conflict events or sexual pursuits (Sueur and Petit, 2008). Simultaneously, behavioral sampling was employed to gather behavioral information for low-probability occurrences (Altmann, 1974), such as *chase*, *bite*, *flee*. We recorded 17 different behaviors (Kohn et al., 2016; Chen R. et al., 2018), among which the observation duration of *sit alone*, *self-groom*, *groom*, and *proximity* were recorded, while the frequency of *self-scratch*, *approach*, *bridge*, *present*, *leave*, *redirection*, *stare*, *ground slap*, *chase*, *seize*, *bite*, *avoid*, and *flee* were recorded. Behavioral definitions are provided in Supplementary Table S2. From February to April 2023, data collection from 24 adult Tibetan macaques yielded 3,600 min of observation and 384 low-probability incidents.

During the analysis, we categorized *bite*, *grasp., stare*, *ground slap*, *redirection* and *chase* as aggressive behaviors, while *avoid* and *flee* were categorized as submissive behaviors (Kohn et al., 2016). Using the principal function from the psych package in R, we performed principal component analysis (PCA) and obtained standardized factor loadings and component scores without employing data rotation (Revelle, 2023). Principal Component Analysis (PCA) was performed on a 24 × 11 matrix of behavioral data, where each row corresponds to a unique individual and each column represents the behavior of interest. The data was normalized by dividing the duration or frequency of individual behaviors by the total duration or frequency of all behaviors. Reliability analyses were also performed using KMO and Bartlett’s test of sphericity, implemented using SPSS 27.0.1. In order to enhance the interpretability of our findings, we utilized both quantitative and qualitative analytical strategies. The qualitative analysis involves grouping the data into high and low quartiles based on scores to explore and interpret the differences in behavioral or functional aspects among these groups. In contrast, the quantitative analysis focused on identifying and quantifying the correlational relationships among the scores, aiming to elucidate the numerical patterns and trends that may exist between the variables under investigation (Park et al., 2021). It should be noted here that because the gut microbiome usually variations in a gradient among social individuals, it is difficult to fully reflect the relationship between personality and gut microbiome by simple personality grouping, so the personality evaluation method used in the present study is different from the previous method used by our research team, but it does not mean that there is a contradiction between the two assessment results.

### 2.4 Bioinformatics and statistical analysis

Using the sliding window approach implemented in fastp v0.19.6, we cropped the raw FASTQ sequencing data for adapter sequences and quality control (Chen S. et al., 2018). Sequences containing N bases were removed. We utilized FLASH (v1.2.7) to merge overlapping paired-end reads, assembling sequences longer than 10 bp based on their overlapped sequence, with a maximum mismatch ratio of 0.2 in the overlap region and discarding unassembleable reads (Magoc and Salzberg, 2011). Furthermore, using DADA2 within Qiime 2 to truncate forward and reverse reads, denoise the data, and identify and eliminate chimeras, we clustered the quality-check of sequences into Amplicon Sequence Variants (ASVs) (Callahan et al., 2016; Bolyen et al., 2019). Classify-sklearn (Naive Bayes) was used to assign taxonomy to Amplicon Sequence Variations using SILVA ribosomal RNA database.^1^ During the analysis, none of the families and genera that were shown as “unclassified” or “norank” in the database were shown in the results.

The Shannon diversity index, Chao 1 index, PD index (phylogenetic diversity index), and weighted and unweighted UniFrac distance matrices were calculated using Qiime 2 (Bolyen et al., 2019). We performed all subsequent analyses on taxa whose mean relative abundance was greater than 0.01% and found in at least 10% of the samples (Kim et al., 2018). The Kolmogorov–Smirnov normality test was utilized in our analysis to assess the normal distribution of relative abundance of bacterial colonies, functional guilds, and alpha diversity index. In addition, we took advantage of the Reconstruction of Unobserved States (version 2; PICRUSt 2), predicting the KEGG pathway by normalizing ASVs by 16S rRNA copy number to gain insight into the function and trophic patterns of bacterial communities (Langille et al., 2013).

To explore whether the composition of the gut microbiome and alpha diversity differed between personality subgroups, we analyzed using an unpaired student’s *t-*test in the case of a normal distribution and the Mann–Whitney *U* test in the case of a non-normal distribution in the qualitative analysis. Principal coordinates analysis (PCoA) was performed with the R packages Made4 and Vegan (Culhane et al., 2005). Permutational multivariate analysis of variance (PERMANOVA) was employed to investigate the differences in beta diversity among individuals with varying personality traits. For identifying species and functional guilds with significant differences between subgroups, the trans_diff function in the microeco package in R was used to implement linear discriminant analysis effect size (LEfSe) (Liu et al., 2021). In this analysis, method is specified as ‘lefse’ and the group variable is set for the groups to be compared, while no method is set to correct for *p*-values. Finally, taxa and functional guilds with LDA values greater than 2 and *p*-values less than 0.05 were used for further analysis.

In the quantitative analysis, the correlation between standardized factor scores derived from the principal component analysis and alpha diversity using a general linear model, controlling for age and gender. The analyses were carried out using the lmer function from the lme4 package in R (Bates et al., 2015). To study the complex relationships between personality traits and microbiome, we used Maaslin2 (Multivariable Association with Linear Models) package of R to implement a generalized linear model (Mallick et al., 2021). MaAslin2 was used to detect the association between microbial composition and each participant’s personality, taking into account the age and sex of the participants, and all analyses used the default options. We considered age and gender together with the standardized factor scores as independent variables, while the alpha diversity index was set as the dependent variable for analysis. To maintain a stringent threshold for statistical significance, the Benjamini–Hochberg correction (FDR) was applied to correct for *p*-values at *q* < 0.1 (Johnson, 2020).

## 3 Results

### 3.1 General patterns of the fecal bacterial profile

After quality filtering, a total of 4,464,436 high-quality readings were obtained from the 94 samples, with an average of 47,494 ± 7,779 sequences per sample. Representatives from 19 known bacterial phyla were found by taxonomic identification at 97% sequence identity, which was dominated by Firmicutes (*x* = 55.04 ± 10.26%) and Bacteroidetes (*x* = 24.80 ± 9.05%). Other phyla represented were Spirochaetota (*x* = 9.50 ± 6.79%), Proteobacteria (*x* = 4.64 ± 4.37%). The dominant families were Oscillospiraceae (*x* = 11.96 ± 3.67%), Prevotellaceae (*x* = 11.31 ± 6.37%), Lachnospiraceae (*x* = 11.11 ± 4.71%). The predominant known genera detected in the fecal samples were *Treponema* (*x* = 8.81 ± 6.78%), *Prevotella* (*x* = 8.66 ± 5.69%).

### 3.2 Personality traits of all study subjects

We evaluated the behavioral dataset for all individuals using principal component analysis (PCA). The results showed that the three principal components (PCs) met the criteria of the loadings plot test with eigenvalues greater than 1 and cumulatively explained 66.396% of the variance in the correlation matrix (Supplementary Figure S1). In addition, because the absolute value of the loading coefficients on the three principal components for all behavioral variables except *present* exceeded 0.5, *present* was not included in the subsequent analysis. The PCA component loadings of different behaviors were presented in Table 1. After analyzing the specific behavioral patterns associated with each component, we identified personality traits that corresponded to the varying scores of individuals on these components. Individuals with higher scores on Component 1 had a reduced inclination to be alone and frequently engaged in social behaviors, including *approach*, *bridge*, *leave*, and *groom*. Consequently, they were classified as “Social.” On the other hand, individuals with higher scores in Component 2 tended to avoid contact with other individuals and rarely exhibited aggressive behavior, leading to the categorization of these individuals as “Shy.” Additionally, those with higher scores on Component 3 displayed a higher frequency of self-grooming and self-scratching behaviors, which are known to be positively correlated with anxiety levels (Zhang et al., 2014). Therefore, this trait was named “Anxious.” Supplementary Table S3 provides the principal component scores for adult individuals of the YA1 group, which will be used for subsequent quantitative analysis.

**Table 1:** Principal component analysis component loadings in adult Tibetan macaques.

Behavior	Components and loadings		
	PC1	PC2	PC3
Sit alone	−0.892^*^	−0.346	0.174
Grooming	0.585^*^	0.536	−0.375
Proximity	0.825^*^	−0.263	−0.218
Bridging	0.713^*^	−0.26	−0.05
Approach	0.689^*^	0.044	0.169
Leave	0.678^*^	0.04	−0.071
Submission	−0.122	0.766^*^	0.198
Aggression	0.544	−0.601^*^	0.1
Present	0.063	0.452	−0.475^*^
Self-grooming	0.191	0.33	0.773^*^
Self-scratching	0.593	0.086	0.631^*^

^*^The heaviest loadings for each item.

Given the complex and variable nature of individual personality, representing them with a single dimension may not fully capture the actual situation. Consequently, individuals were divided into positive and negative groups based on their scores on principal components (positive group: above the lower quartile; negative group: below the upper quartile). To better differentiate between groups, distinct names are assigned to the positive and negative groups for each principal component. For instance, individuals in the positive Sociality group tend to engage in social interactions such as grooming, approaching, and bridging, and are thus named “Sociality.” Conversely, individuals in the negative group are more likely to avoid social interactions and prefer solitude, and are named “Loneliness.” By extension, the remaining subgroups are named “Shyness” and “Boldness,” as well as “Anxiety” and “Calmness.” This categorization will be employed for subsequent qualitative analysis.

### 3.3 Gut microbial diversity and personality traits

**Figure 1:**
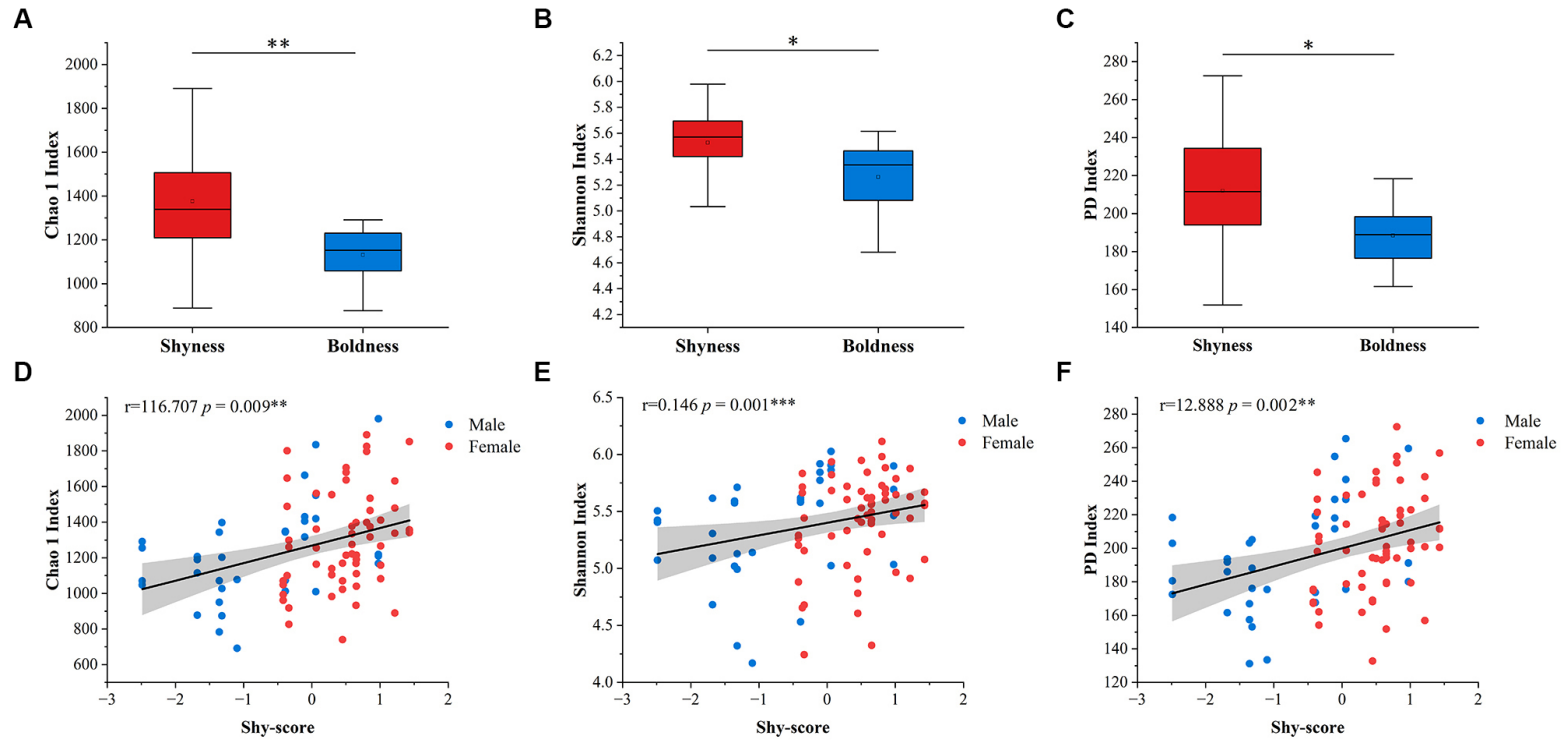
Variation of alpha diversity in the dimensions of shyness. Chao 1 index, Shannon index, and PD index were used for alpha diversity. **(A–C)** represent qualitative traits; **(D–F)** represent quantitative traits. The sample size and detailed results are shown in Supplementary Tables S4, S5. Shaded areas represent 95% confidence intervals. ^*^p-value < 0.05, ^**^p-value < 0.01, ^***^p-value < 0.001.

Considering age and sex, only the Shy Dimension exhibits a correlation with alpha diversity indices (Shannon: *p* = 0.008; Chao 1: *p* = 0.001; PD: *p* = 0.001, General linear model, Figure 1A–C), while other personality dimensions do not show significant correlations with alpha diversity (Supplementary Table S4). Our qualitative analysis further supports these findings. We consistently observed that the alpha diversity indices of the Shyness are significantly higher than those of the Boldness (Shannon: *p* = 0.016; Chao 1: *p* = 0.004, Mann–Whitney *U* test; PD: *p* = 0.001, unpaired student’s *t-*test, Figure 1D–F). No significant differences were found among the groups of other personality types (Supplementary Figure S2). In addition, gender had no significant effect on the relationship between personality and alpha diversity.

**Figure 2:**
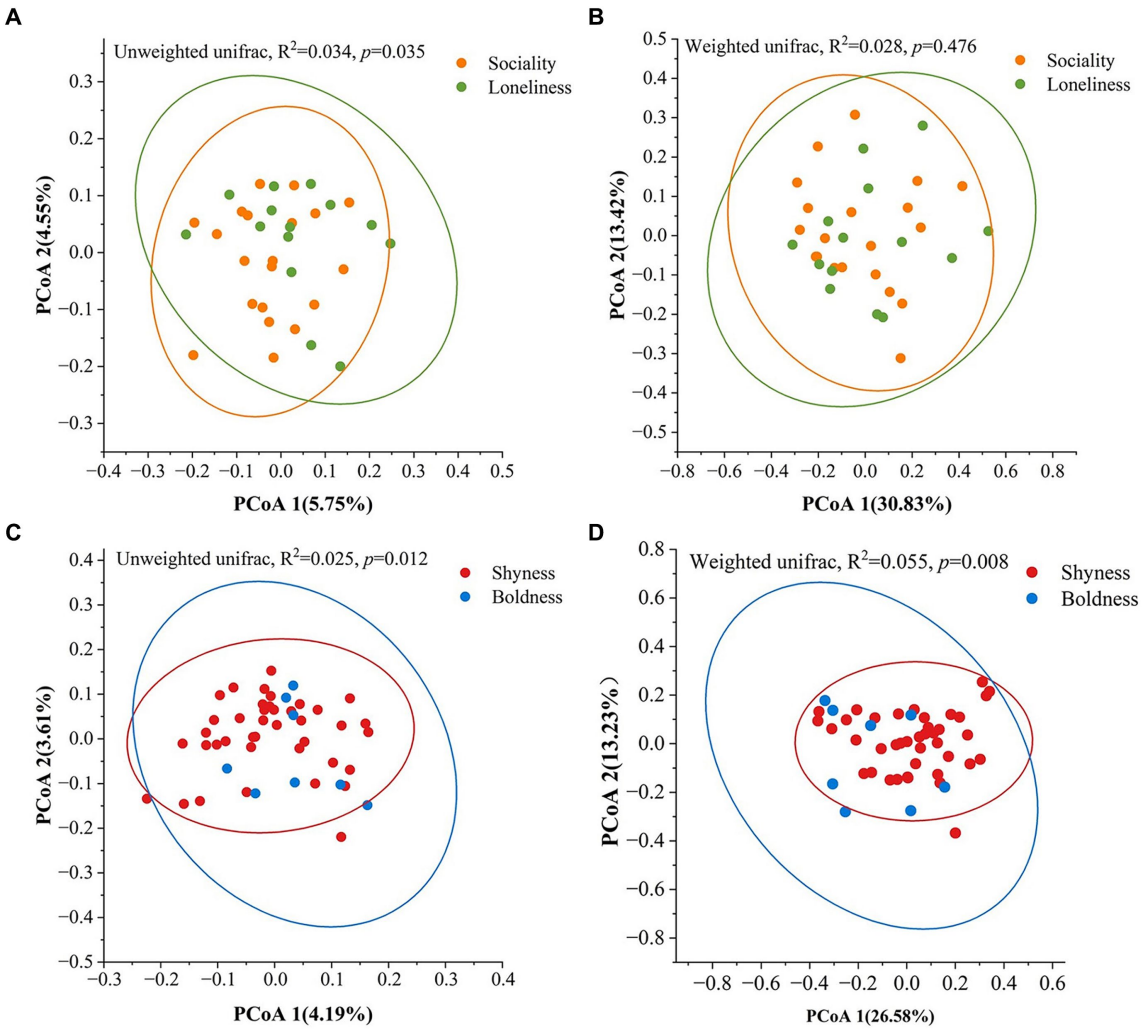
Variation of beta diversity among different personality groups. **(A,C)** Based on unweighted UniFrac distance. **(B,D)** Based on weighted UniFrac distance. The ellipses represent a 95% confidence interval for each group. Significance was set at the 0.05 level.

We conducted PCoA and PERMANOVA tests on weighted and unweighted UniFrac distances to investigate the prediction of beta diversity by different personality dimensions. The PERMANOVA tests revealed significant separation between sociality and loneliness based on unweighted UniFrac distances (*R*^2^ = 0.034, *p* = 0.035, Figure 2A), whereas no significant separation was detected when employing the weighted distances (*R*^2^ = 0.028, *p* = 0.476, Figure 2B). In addition, a significant divergence was observed between shyness and boldness (unweighted UniFrac, *R*^2^ = 0.024, *p* = 0.012; weighted UniFrac, *R*^2^ = 0.054, *p* = 0.008, Figures 2C,D). However, no significant divergence was detected between anxiety and calmness (Supplementary Figure S3).

### 3.4 Correlations of gut microbial composition and personality scores

**Figure 3:**
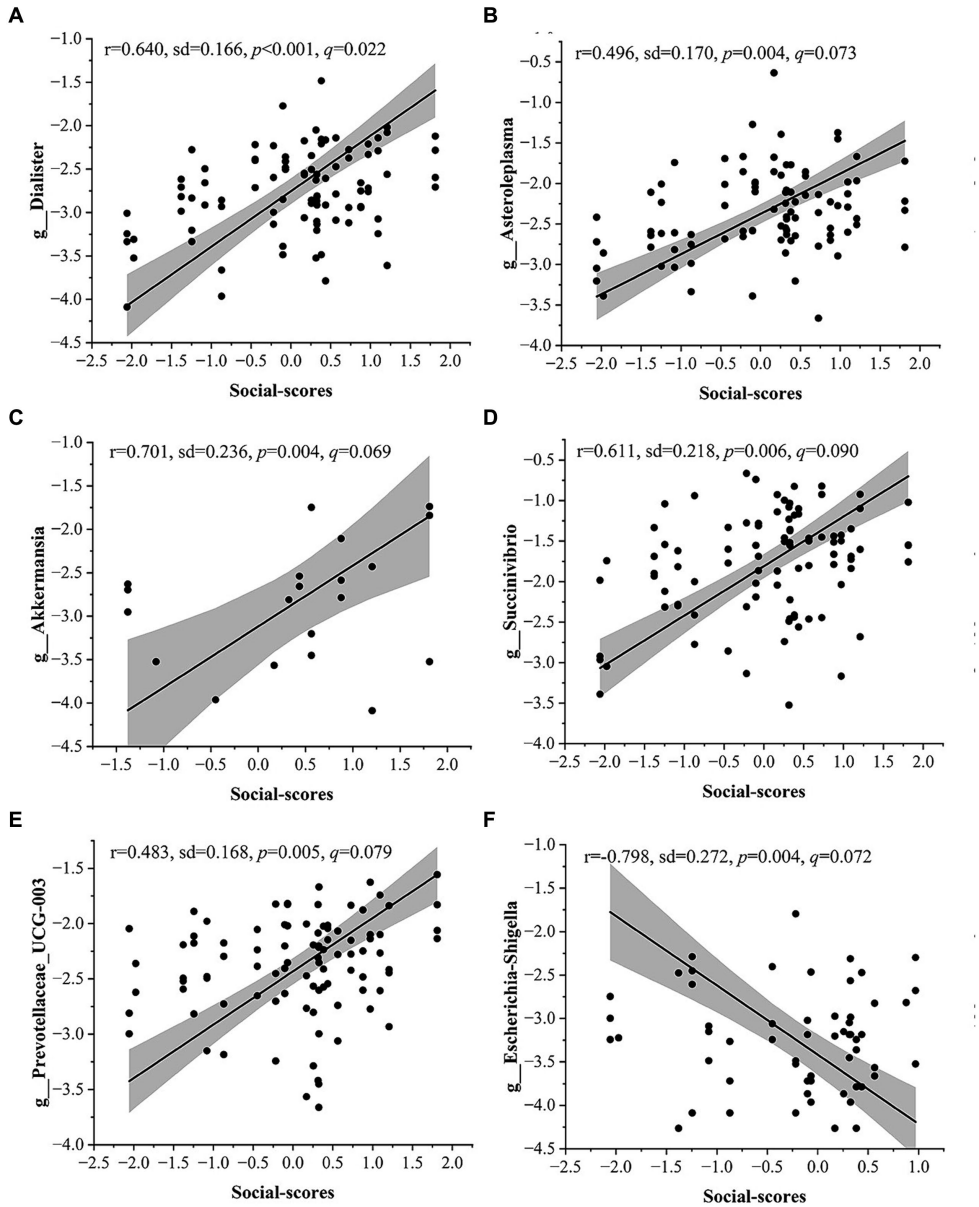
Correlation between gut microbiota and personality traits in Maaslin2 analysis. **(A-F)** Genus-level taxa are significantly correlated with sociability. The relative abundance of all bacteria was normalized (Log) and the sample size was 94 (the relative abundance of 0 is not shown in the figure). The taxon of *p* < 0.05 and *q* < 0.1 is listed. Shaded areas represent 95% confidence intervals.

We performed MaAsLin2 analyses to identify specific microbial taxa significantly associated with personality traits, taking into account age and gender. We performed domain-specific analyses for each personality trait and there were 12 candidate taxa significantly associated with personality traits (false discovery rate of *q* < 0.1, Figure 3A–F and Supplementary Table S6). Among them, nine taxa exhibited a significant positive correlation with factor scores for Social, including Veillonellaceae (*r* = 0.701, *p* < 0.001, *q* = 0.010), Akkermansiaceae (*r* = 0.701, *p* = 0.004, *q* = 0.069), Succinivibrionaceae (*r* = 0.611, *p* = 0.006, *q* = 0.089), Desulfovibrionaceae (*r* = 0.666, *p* < 0.001, *q* = 0.031), *Dialister* (*r* = 0.640, *p* < 0.001 *q* = 0.022), *Prevotellaceae_UCG.003* (*r* = 0.483, *p* = 0.005, *q* = 0.079), *Akkermansia* (*r* = 0.701, *p* = 0.004, *q* = 0.069), *Asteroleplasma* (*r* = 0.496, *p* = 0.004, *q* = 0.073), *Succinivibrio* (*r* = 0.611, *p* = 0.006, *q* = 0.090). Two taxa were significantly negatively correlated with the scores for Social Dimension, including Enterobacteriaceae (*r* = −0.811, *p* = 0.004, *q* = 0.069) and *Escherichia-Shigella* (*r* = −0.798, *p* = 0.004, *q* = 0.072). Furthermore, Oscillospiraceae (*r* = 0.222, *p* = 0.001, *q* = 0.073) exhibited a positive correlation with factor scores for Shy Dimension.

### 3.5 Variation of microbial composition among different personality groups

**Figure 4:**
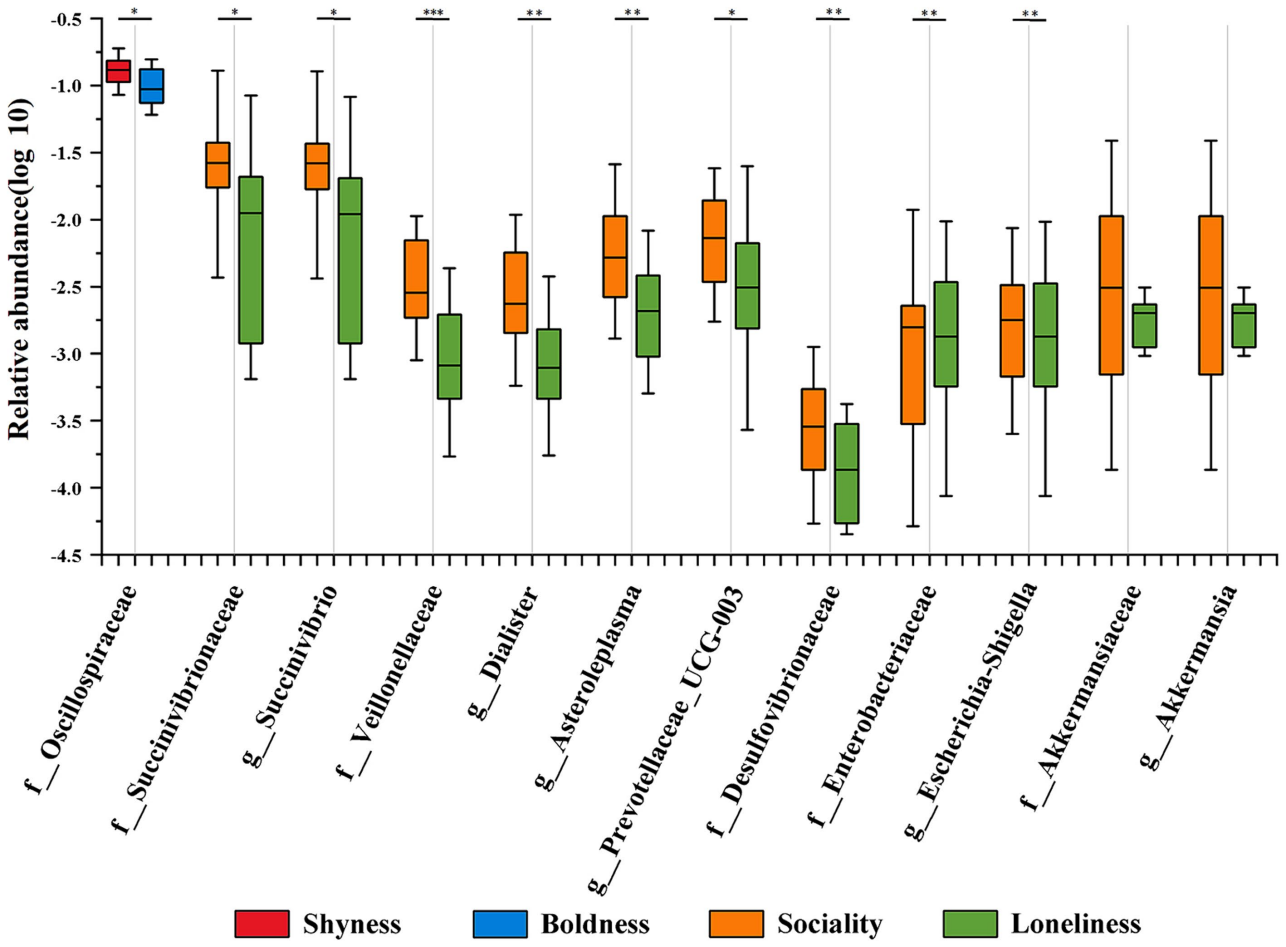
Differential distribution of personality-associated taxa in Maaslin2 analysis across personality groups. Different colors represent different personality groups. The median line of each box plot is the median. The upper and lower whiskers represent the standard deviation. ^*^p-value < 0.05, ^**^p-value < 0.01.

In order to gain a deeper understanding of the results obtained from the Maaslin2 analysis, a pairwise difference comparison (negative group vs. positive group of a specific category of personality) was performed on the microbial taxa obtained by MaAsLin2 analysis. The results revealed distinctions among the 10 identified taxonomic groups in their respective cohorts, aligning with the conclusions drawn from MaAsLin2 analysis (Figure 4 and Supplementary Table S7). Seven taxonomic groups, including Veillonellaceae (*p* = 0.001), *Dialister* (*p* = 0.003), Desulfovibrionaceae (*p* = 0.004), Succinivibrionaceae (*p* = 0.027), *Prevotellaceae*_UCG.003 (*p* = 0.036), *Asteroleplasma* (*p* = 0.006), and *Succinivibrio* (*p* = 0.025), were significantly more abundant in the sociality group compared to the loneliness group. In contrast, the loneliness group had a higher abundance of two taxonomic groups, Enterobacteriaceae (*p* = 0.017) and *Escherichia-Shigella* (*p* = 0.014). Additionally, Oscillospiraceae (*p* = 0.040) was significantly more abundant in the shyness group than in the boldness group. However, it is noteworthy that although the mean relative abundance of Akkermansiaceae and *Akkermansia* tends to be higher in the sociality group compared to the loneliness group, this difference is not statistically significant (*p* > 0.05).

**Figure 5:**
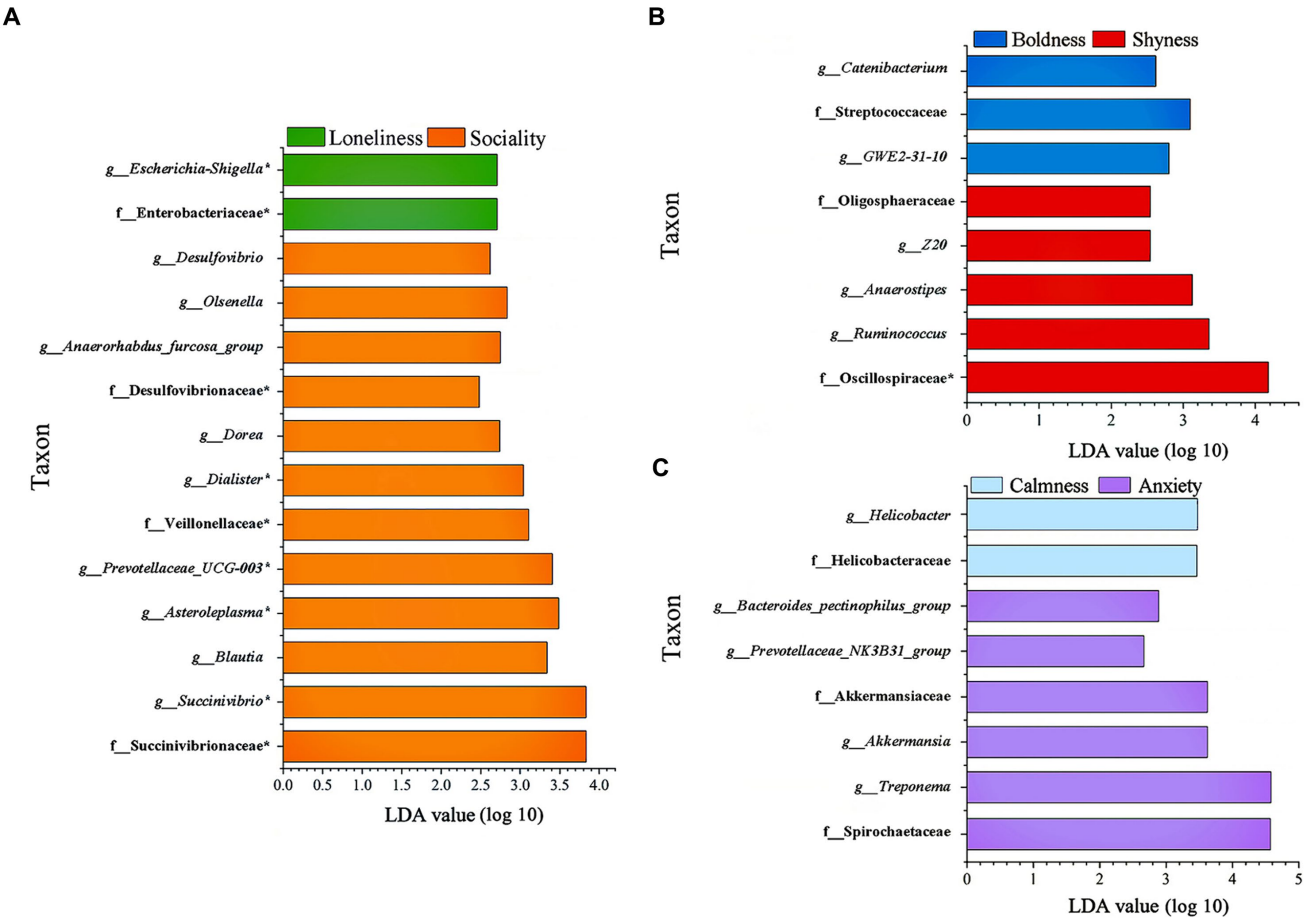
Indicators of known bacterial taxa among different personality traits. **(A)** Sociality and Loneliness, **(B)** Shyness and Boldness, **(C)** Anxiety and Calmness. f_ family; g_ genus, identified by linear discriminant analysis effect size (LEfSe) analysis (LDA > 2, *p* < 0.05).

In addition, LEfSe analysis was employed to pinpoint significant variations in biomarkers at the family and genus levels across different groups (LDA > 2, *p* < 0.05, Figure 5). In the sociality group, three families including Veillonellaceae, Desulfovibrionaceae, and Succinivibrionaceae, and nine genera including *Desulfovibrio*, *Olsenella*, *Anaerorhabdus_furcosa_group*, *Prevotellaceae_UCG-003*, *Dorea*, *Dialister*, *Asteroleplasma*, *Blautia*, and *Succinivibrio* were prominently enriched. Conversely, Enterobacteriaceae and *Escherichia-Shigella* were found to be enriched in the loneliness group. The shyness group had an enrichment of five taxonomic groups, including two families (Oscillospiraceae and Oligosphaeraceae) and three genera (*Anaerostipes*, *Ruminococcus* and *Z20*). In the boldness group, one family (Streptococcaceae) and two genera (*Catenibacterium* and *GWE2-31-10*) were enriched. The anxiety group was characterized by the enrichment of two families, (Spirochaetaceae and Akkermansiaceae), as well as four genera (*Treponema, Bacteroides_pectinophilus_group*, *Prevotellaceae_NK3B31_group,* and *Akkermansia*). Two taxa (Helicobacteraceae, *Helicobacter*) were enriched in the calmness group.

### 3.6 Predicted functional metagenome in personality groups

**Figure 6:**
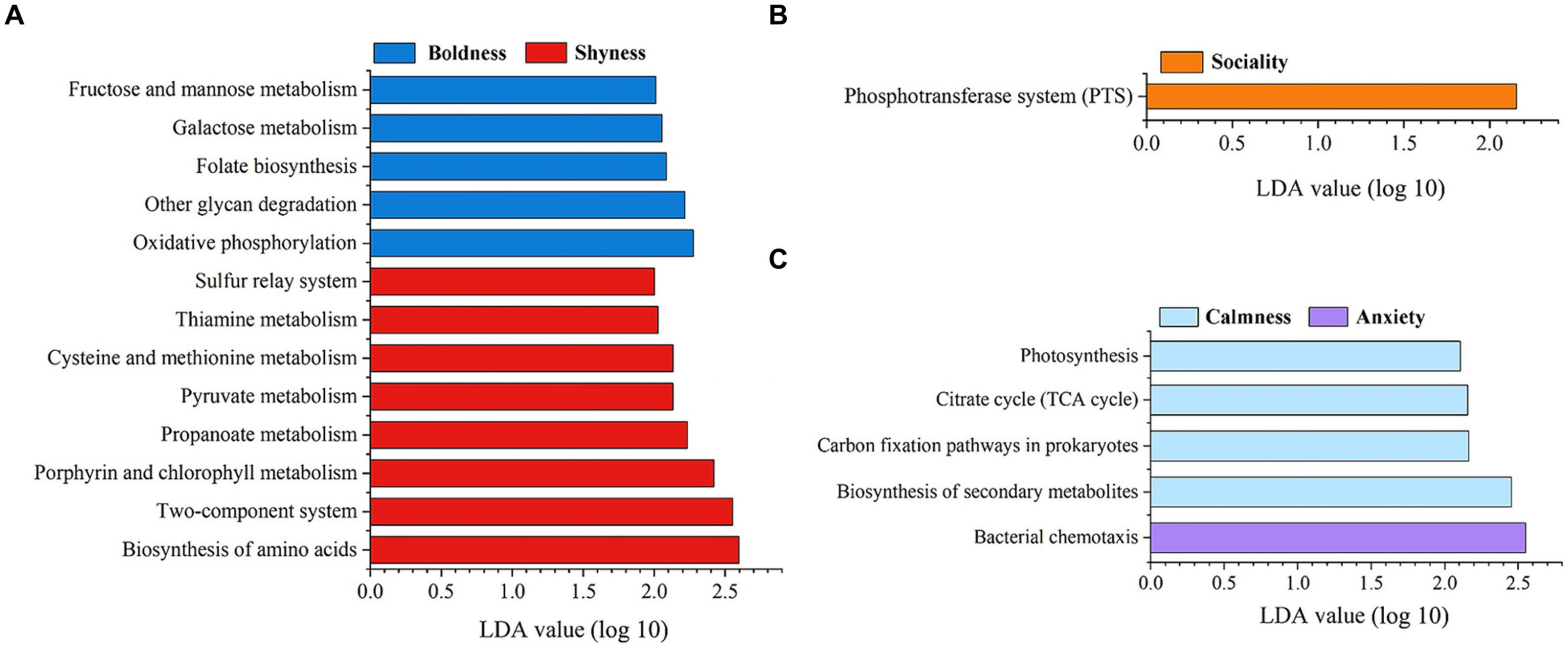
Predicts personality-related functional content enriched in different personality traits. **(A)** Shyness and Boldness, **(B)** Sociality and Loneliness, **(C)** Anxiety and Calmness. The KEGG pathway is used in the KEGG-level-3 category. The predicted functional genes enriched in different groups were identified by LEfSe analysis (LDA > 2, *p* < 0.05).

Furthermore, LEfSe analyses showed that several predicted metabolic functions (KEGG pathway level 3) were enriched in one of the two groups for specific personality categories (LDA > 2, *p* < 0.05). The predicted functional genes of Phosphotransferase system were overrepresented in the Sociality individuals compared to the Loneliness subtype (Figure 6A). Additionally, five (Oxidative phosphorylation, Folate biosynthesis, Galactose metabolism, Fructose and mannose metabolism, and Other glycan degradation) and eight (Biosynthesis of amino acids, Two-component system, Porphyrin and chlorophyll metabolism, Propanoate metabolism, Pyruvate metabolism, Cysteine and methionine metabolism, Thiamine metabolism, and Sulfur relay system) KEGG pathways were enriched in Boldness and Shyness groups, respectively, (Figure 6B). Concurrently, four KEGG pathways (Biosynthesis of secondary metabolites, Carbon fixation pathways in prokaryotes, Citrate cycle, and Photosynthesis) were overrepresented in the group of Calmness individuals. Only the metabolic pathway of Bacterial chemotaxis was detected to be enriched in Anxiety group (Figure 6C).

## 4 Discussion

In the current study, we found differences in alpha and beta diversity as well as the specific bacterial taxa and predicted functional metagenome of the gut microbiome of different personality traits in Tibetan macaques. Previous studies in human and laboratory animals have shown that personality can predict the diversity of gut microbiome. For example, individuals in early childhood with high alpha diversity tend to have higher extraversion (Christian et al., 2015; Jiang et al., 2015), and low alpha diversity is strongly associated with increased expression of negative emotions in human infants (Kelsey et al., 2021). However, a recent study did not find significant differences in the alpha and beta diversity of the gut microbiome between bold and shy Mongolian gerbils (*Meriones unguiculatus*) (Gan et al., 2022). Our findings showed that alpha diversity is lower in boldness individuals than in shyness individuals on the Shy Dimension. Individuals with boldness personality typically show higher levels of aggression in Tibetan macaques. Previous study in this species has shown that adult males with higher aggressive behavior also have higher cortisol levels than those of low-ranked males (Wu et al., 2021), implying that individuals with higher levels of aggressive behavior may face higher stress levels. Furthermore, increased social stress has also been shown to lead to a decrease in the diversity of gut microbiome (Bailey et al., 2011; Partrick et al., 2018). This may offer a possible explanation for boldness individuals had lower gut microbial diversity than shyness individuals.

In addition, recent studies have shown that the enrichment of some specific microbial taxa in the gut can influence the behavior and personality of animals. For example, there is a significant positive correlation between the relative abundance of *Akkermansia* and human sociability (Johnson, 2020). In autistic spectrum disorder (ASD) patients, the relative abundance of *Akkermansia* and *Dialister* was significantly lower in depressed patients compared to non-depressed patients (Valles-Colomer et al., 2019; Yang et al., 2020). Consistent with previous studies, we also found that individuals with higher sociability scores in Tibetan macaques had higher relative abundance of *Akkermansia* and *Dialister* in their gut. Both genera are involved in carbohydrate metabolism and produce short-chain fatty acids (SCFAs) such as acetate, propionate and butyrate (Jiang et al., 2015; Morris et al., 2017). In addition, butyrate and other by-products of microbial fermentation have been reported as chemical messengers that promote social interaction (Stilling et al., 2016). Moreover, *Akkermansia* has been shown to increase levels of serotonin (5-HT), which is thought to improve socialization in humans (Walsh et al., 2018; Guo et al., 2023). On the other hand, *Asteroleplasma* is positively correlated with communicative abilities, which may be related to its promotion of α-ketoglutarate production (Gu et al., 2021). Since α-ketoglutarate serves as a precursor to glutamate, a neurotransmitter known to facilitate social play in adolescents, *Asteroleplasma* may enhance social interaction by increasing the availability of glutamate (Moussawi et al., 2011; Jo et al., 2012; Bredewold et al., 2015). Thus, our study suggests that the increase of *Akkermansia*, *Dialister* and *Asteroleplasma* in the gut microbiome of Tibetan macaques may contribute to the personality traits of sociality.

Although *Ruminococcus* was not identified in the regression analysis for Shy Dimension, it was significantly enriched in individuals of shyness group. In addition, it was shown that the relative abundance of *Ruminococcus* was high in the gut flora of Tibetan macaques, prompting us to consider that it may have an important link to host behavior. Research on the personality of infants aged between 1 and 12 months has found that *Ruminococcus* is positively associated with negative emotions, including sadness and fear, at 12 months of age (Fox et al., 2021). In addition, *Ruminococcus* has also been found to be associated with depression and negative emotions in research on brain health and disease (Coretti et al., 2019; Ahmed et al., 2020; Wang et al., 2020). In Tibetan macaques, individuals of the shyness group tend to avoid others and experience more attacks from within the group, with a greater probability of negative emotions. This may explain the significant abundance of *Ruminococcus* in individuals of the shyness group. In contrast, Oscillospiraceae, another group of bacteria, has been demonstrated to be enriched in depression using various depression models (Liu et al., 2022). However, no significant association has been reported between this family and traits of shyness or boldness, and its potential role on personality needs to be further explored and researched.

Furthermore, we found that predicted functional genes for several metabolic pathways of gut microbiome, related to animal behavior and personality, were enriched across personality types. The boldness group showed a significant enrichment of metabolic pathways for fructose, mannose, and galactose, leading to elevated blood glucose levels (Kovacic and Somanathan, 2013; Patel et al., 2015). Studies have shown that hyperglycemic state is closely associated with insulin resistance (Brown and Goldstein, 2008; Huri et al., 2014). Insulin resistance is positively correlated with elevated cortisol levels (Lehrke et al., 2008; Nguyen et al., 2023), which have been shown to facilitate an increase in aggressive behaviors (Alink et al., 2008; Azurmendi et al., 2016). This could be a significant contributing factor to the more frequent aggressive behavior observed in the boldness group of Tibetan macaques. Additionally, our result revealed a significant enrichment of propionate and pyruvate metabolic pathways within the boldness group. It is noteworthy that propionate can be converted into pyruvate through a series of complex biochemical reactions (Wheeler et al., 2008), suggesting a shared functional significance in the enrichment of both pathways. Recent research indicates that experimental rats treated with pyruvate exhibit a significant reduction in aggression, indicating that pyruvate can mitigate host aggressive behavior (Frank et al., 2023), which contributes to the understanding of the lower aggression observed in the shy group. Furthermore, the shyness group was found to exhibit enrichment in porphyrin and chlorophyll metabolic pathways, aligning with prior research outcomes in Mongolian gerbils (Gan et al., 2022).

Although there is evidence that phenylalanine and tyrosine are precursors of a variety of secondary metabolites that inhibit 5-hydroxytryptophan production, which may contribute to anxiety (Millan, 2003; Ruhe et al., 2007; Goni-Allo et al., 2008; Albert et al., 2014), whether the enrichment of secondary metabolite biosynthetic pathways contributes to the development of an anxious personality in Tibetan macaques remains to be investigated. In the field of human health research, the presence of gut bacteria such as *Parabacteroides*, *Bifidobacterium,* and *Faecalibacterium* has been firmly associated with anxiety (Taylor et al., 2020; Simpson et al., 2021). However, due to the limited feasibility of assessing specific anxiety behavior types, compounded by the challenging nature of collecting behavioral data in wild animals, our study failed to identify microbial taxa associated with anxious. Therefore, specialized research with a focus on anxiety is imperative. Moreover, accurately determining factors such as weight, dietary preferences, and consumption rates in wildlife presents significant difficulties in our study, which constitutes a limitation that needs to be addressed.

## 5 Conclusion

In this study, we attempted to explore the relationship between personality traits and gut flora in a wild primate social group. Our findings demonstrate that the personality traits are associated with the differences in alpha and beta diversity of the gut microbiome in Tibetan macaques, as well as the taxonomic composition and prediction of functional genes. Our study suggests that the gut microbiome may play an important role in the formation of personality of Tibetan macaques. However, it will be necessary to demonstrate the specific function of gut flora in the formation of personality traits in non-human primates and the pathways through which they are influenced, in conjunction with fecal bacterial transplantation (FMT) techniques. Although the effects of gender and age were taken into account in this study, other important factors such as food and environment were not addressed. Moreover, limited by existing knowledge, the enrichment of some specific bacterial taxa and predicted functional metagenomes of the gut microbiome in different personality traits of Tibetan macaques is not yet well explained. More research is still needed regarding the relationship between gut microbiome and personality of Tibetan macaques.

## Data availability statement

The datasets presented in this study can be found in online repositories. This data can be found on line at: https://www.ncbi.nlm.nih.gov/bioproject/?term=PRJNA1067856.

## Ethics statement

The animal study was approved by the Institutional Animal Care and Use Committee of the Anhui Zoological Society (permit number BH20221203). The study was conducted in accordance with the local legislation and institutional requirements.

## Author contributions

MX: Data curation, Formal analysis, Investigation, Methodology, Software, Validation, Visualization, Writing – original draft, Writing – review & editing. YX: Data curation, Formal analysis, Investigation, Methodology, Software, Validation, Writing – original draft, Writing – review & editing. YS: Data curation, Formal analysis, Investigation, Methodology, Software, Validation, Writing – review & editing. JW: Data curation, Investigation, Methodology, Software, Validation, Writing – review & editing. JL: Data curation, Investigation, Methodology, Software, Validation, Writing – review & editing, Formal analysis. XW: Formal analysis, Methodology, Software, Validation, Writing – review & editing. DX: Formal analysis, Methodology, Software, Validation, Writing – review & editing. XX: Conceptualization, Data curation, Funding acquisition, Investigation, Project administration, Resources, Supervision, Writing – original draft, Formal analysis, Methodology, Software, Validation, Writing – review & editing. BS: Conceptualization, Data curation, Formal analysis, Funding acquisition, Investigation, Methodology, Project administration, Resources, Software, Supervision, Validation, Writing – original draft, Writing – review & editing, Visualization.

## Footnotes

^1^ https://www.arb-silva.de/

## References

[ref1] Aatsinki A. K. Lahti L. Uusitupa H. M. Munukka E. Keskitalo A. Nolvi S.. (2019). Gut microbiota composition is associated with temperament traits in infants. Brain Behav. Immun. 80, 849–858. doi: 10.1016/j.bbi.2019.05.035, PMID: 31132457

[ref2] Ahmed S. A. Elhefnawy A. M. Azouz H. G. Roshdy Y. S. Ashry M. H. Ibrahim A. E.. (2020). Study of the gut microbiome profile in children with autism Spectrum disorder: a single tertiary hospital experience. J. Mol. Neurosci. 70, 887–896. doi: 10.1007/s12031-020-01500-3, PMID: 32062762

[ref3] Albert P. R. Vahid-Ansari F. Luckhart C. (2014). Serotonin-prefrontal cortical circuitry in anxiety and depression phenotypes: pivotal role of pre- and post-synaptic 5-HT1A receptor expression. Front. Behav. Neurosci. 8:199. doi: 10.3389/fnbeh.2014.00199, PMID: 24936175 PMC4047678

[ref4] Ali H. Muhammad A. Sanda N. B. Huang Y. Hou Y. (2019). Pyrosequencing uncovers a shift in bacterial communities across life stages of *Octodonta nipae* (Coleoptera: Chrysomelidae). Front. Microbiol. 10:466. doi: 10.3389/fmicb.2019.00466, PMID: 30930872 PMC6424052

[ref5] Alink L. R. Van Ijzendoorn M. H. Bakermans-Kranenburg M. J. Mesman J. Juffer F. Koot H. M. (2008). Cortisol and externalizing behavior in children and adolescents: mixed meta-analytic evidence for the inverse relation of basal cortisol and cortisol reactivity with externalizing behavior. Dev. Psychobiol. 50, 427–450. doi: 10.1002/dev.20300, PMID: 18551461

[ref6] Altmann J. (1974). Observational study of behavior: sampling methods. Behaviour 49, 227–266. doi: 10.1163/156853974X00534 4597405

[ref7] Altschul D. M. Hopkins W. D. Herrelko E. S. Inoue-Murayama M. Matsuzawa T. King J. E.. (2018). Personality links with lifespan in chimpanzees. eLife 7:e33781. doi: 10.7554/eLife.33781, PMID: 30296994 PMC6177254

[ref8] Azurmendi A. Pascual-Sagastizabal E. Vergara A. I. Munoz J. M. Braza P. Carreras R.. (2016). Developmental trajectories of aggressive behavior in children from ages 8 to 10: the role of sex and hormones. Am. J. Hum. Biol. 28, 90–97. doi: 10.1002/ajhb.22750, PMID: 26089078

[ref9] Bailey M. T. Dowd S. E. Galley J. D. Hufnagle A. R. Allen R. G. Lyte M. (2011). Exposure to a social stressor alters the structure of the intestinal microbiota: implications for stressor-induced immunomodulation. Brain Behav. Immun. 25, 397–407. doi: 10.1016/j.bbi.2010.10.023, PMID: 21040780 PMC3039072

[ref10] Barandouzi Z. A. Lee J. Rosas M. D. C. Chen J. Henderson W. A. Starkweather A. R.. (2022). Associations of neurotransmitters and the gut microbiome with emotional distress in mixed type of irritable bowel syndrome. Sci. Rep. 12:1648. doi: 10.1038/s41598-022-05756-0, PMID: 35102266 PMC8803858

[ref11] Bates D. Maechler M. Bolker B. M. Walker S. C. (2015). Fitting linear mixed-effects models using lme4. J. Stat. Softw. 67, 1–48. doi: 10.18637/jss.v067.i01

[ref12] Blaszczyk M. B. (2020). Primates got personality, too: toward an integrative primatology of consistent individual differences in behavior. Evol. Anthropol. 29, 56–67. doi: 10.1002/evan.21808, PMID: 31721372

[ref13] Bolyen E. Rideout J. R. Dillon M. R. Bokulich N. Abnet C. C. Al-Ghalith G. A.. (2019). Reproducible, interactive, scalable and extensible microbiome data science using QIIME 2. Nat. Biotechnol. 37, 852–857. doi: 10.1038/s41587-019-0209-9, PMID: 31341288 PMC7015180

[ref14] Brandao A. Costa R. Rodrigues E. Vicente L. (2019). Using behaviour observations to study personality in a group of capuchin monkeys (*Cebus apella*) in captivity. Behaviour 156:243. doi: 10.1163/1568539X-00003537

[ref15] Bredewold R. Schiavo J. K. Van Der Hart M. Verreij M. Veenema A. H. (2015). Dynamic changes in extracellular release of GABA and glutamate in the lateral septum during social play behavior in juvenile rats: implications for sex-specific regulation of social play behavior. Neuroscience 307, 117–127. doi: 10.1016/j.neuroscience.2015.08.052, PMID: 26318330 PMC4591248

[ref16] Brown M. S. Goldstein J. L. (2008). Selective versus total insulin resistance: a pathogenic paradox. Cell Metab. 7, 95–96. doi: 10.1016/j.cmet.2007.12.009, PMID: 18249166

[ref17] Callahan B. J. Mcmurdie P. J. Rosen M. J. Han A. W. Johnson A. J. A. Holmes S. P. (2016). DADA2: high-resolution sample inference from Illumina amplicon data. Nat. Methods 13, 581–583. doi: 10.1038/nmeth.3869, PMID: 27214047 PMC4927377

[ref18] Chen R. Gu Z. Wang X. Sun B. Xia D. Jinhua L. (2018). Personality and its differences among adult free-ranging Tibetan macaques (*Macaca thibetana*) at Mt. Huangshan, China. Acta Theriol Sin. 38, 117–127. doi: 10.16829/j.slxb.150101

[ref19] Chen S. Zhou Y. Chen Y. Gu J. (2018). Fastp: an ultra-fast all-in-one FASTQ preprocessor. Bioinformatics 34, i884–i890. doi: 10.1093/bioinformatics/bty560, PMID: 30423086 PMC6129281

[ref20] Chidambaram S. B. Rathipriya A. G. Mahalakshmi A. M. Sharma S. Hediyal T. A. Ray B.. (2022). The influence of gut dysbiosis in the pathogenesis and Management of Ischemic Stroke. Cells 11:1239. doi: 10.3390/CELLS11071239, PMID: 35406804 PMC8997586

[ref21] Christian L. M. Galley J. D. Hade E. M. Schoppe-Sullivan S. Dush C. K. Bailey M. T. (2015). Gut microbiome composition is associated with temperament during early childhood. Brain Behav. Immun. 45, 118–127. doi: 10.1016/j.bbi.2014.10.018, PMID: 25449582 PMC4342262

[ref22] Coretti L. Paparo L. Riccio M. P. Amato F. Cuomo M. Natale A.. (2019). Corrigendum: gut microbiota features in young children with autism Spectrum disorders. Front. Microbiol. 10:920. doi: 10.3389/fmicb.2019.00920, PMID: 31130927 PMC6509565

[ref23] Cryan J. F. O'riordan K. J. Cowan C. S. M. Sandhu K. V. Bastiaanssen T. F. S. Boehme M.. (2019). The microbiota-gut-brain Axis. Physiol. Rev. 99, 1877–2013. doi: 10.1152/physrev.00018.2018 31460832

[ref24] Culhane A. C. Thioulouse J. Perrière G. Higgins D. G. (2005). MADE4: an R package for multivariate analysis of gene expression data. Bioinformatics 21, 2789–2790. doi: 10.1093/bioinformatics/bti394, PMID: 15797915

[ref25] Davidson G. L. Cooke A. C. Johnson C. N. Quinn J. L. (2018). The gut microbiome as a driver of individual variation in cognition and functional behaviour. Philos. Trans. R. Soc. Lond. Ser. B Biol. Sci. 373:20170286. doi: 10.1098/rstb.2017.0286, PMID: 30104431 PMC6107574

[ref26] Dinuzzo E. R. Griffen B. D. (2020). The effects of animal personality on the ideal free distribution. Proc. Royal Soc. B. 287:20201095. doi: 10.1098/rspb.2020.1095, PMID: 32873202 PMC7542775

[ref27] Fox M. Lee S. M. Wiley K. S. Lagishetty V. Sandman C. A. Jacobs J. P.. (2021). Development of the infant gut microbiome predicts temperament across the first year of life. Dev. Psychopathol. 34, 1914–1925. doi: 10.1017/S0954579421000456, PMID: 34108055 PMC9463039

[ref28] Frank D. Gruenbaum B. F. Shelef I. Zvenigorodsky V. Severynovska O. Fleidervish I.. (2023). Blood glutamate scavenging as a novel glutamate-based therapeutic approach for post-traumatic brain injury anxiety and social impairment. Transl. Psychiatry 13:41. doi: 10.1038/S41398-023-02329-1, PMID: 36739271 PMC9899234

[ref29] Gan L. Bo T. Liu W. Wang D. (2022). The gut microbiota may affect personality in Mongolian gerbils. Microorganisms 10:1054. doi: 10.3390/MICROORGANISMS10051054, PMID: 35630496 PMC9146877

[ref30] Gartner M. C. Powell D. (2012). Personality assessment in snow leopards (*Uncia uncia*). Zoo Biol. 31, 151–165. doi: 10.1002/zoo.20385, PMID: 21455952

[ref31] Goni-Allo B. Puerta E. Mathuna B. O. Hervias I. Lasheras B. De La Torre R.. (2008). On the role of tyrosine and peripheral metabolism in 3,4-methylenedioxymethamphetamine-induced serotonin neurotoxicity in rats. Neuropharmacology 54, 885–900. doi: 10.1016/j.neuropharm.2008.01.007, PMID: 18329670

[ref32] Gu F. Liang S. Zhu S. Liu J. Sun H.-Z. (2021). Multi-omics revealed the effects of rumen-protected methionine on the nutrient profile of milk in dairy cows. Food Res. Int. 149:110682. doi: 10.1016/J.FOODRES.2021.110682, PMID: 34600684

[ref33] Guo H. Liu X. Chen T. Wang X. Zhang X. (2023). *Akkermansia muciniphila* improves depressive-like symptoms by modulating the level of 5-HT neurotransmitters in the gut and brain of mice. Mol. Neurobiol. 61, 821–834. doi: 10.1007/s12035-023-03602-6, PMID: 37668965 PMC10861622

[ref34] Hancock J. M. Vieira S. Taraveira L. Santos A. Schmitt V. Semedo A.. (2019). Genetic characterization of green turtles (*Chelonia mydas*) from Sao Tome and Principe: insights on species recruitment and dispersal in the Gulf of Guinea. J. Exp. Mar. Biol. Ecol. 518:151181. doi: 10.1016/j.jembe.2019.151181

[ref35] Huri H. Z. Makmor-Bakry M. Hashim R. Mustafa N. Ngah W. Z. W. (2014). A prospective cohort study on IRS gene polymorphisms in type 2 diabetes mellitus patients during severe/acute Hyperglycemia phase 1: association with insulin resistance. Trop. J. Pharm. Res. 13, 895–901. doi: 10.4314/tjpr.v13i6.10

[ref36] Jiang H. Ling Z. Zhang Y. Mao H. Ma Z. Yin Y.. (2015). Altered fecal microbiota composition in patients with major depressive disorder. Brain Behav. Immun. 48, 186–194. doi: 10.1016/j.bbi.2015.03.016, PMID: 25882912

[ref37] Jo J.-H. Seol H.-Y. Lee Y.-B. Kim M.-H. Hyun H.-H. Lee H.-H. (2012). Disruption of genes for the enhanced biosynthesis of α-ketoglutarate in *Corynebacterium glutamicum*. Can. J. Microbiol. 58, 278–286. doi: 10.1139/w11-132, PMID: 22356563

[ref38] Johnson K. V. A. (2020). Gut microbiome composition and diversity are related to human personality traits. Hum. Microbiome J. 15:100069. doi: 10.1016/j.humic.2019.100069, PMID: 34435164 PMC8336012

[ref39] Kelly J. R. Borre Y. O' Brien C. Patterson E. el Aidy S. Deane J.. (2016). Transferring the blues: depression-associated gut microbiota induces neurobehavioural changes in the rat. J. Psychiatr. Res. 82, 109–118. doi: 10.1016/j.jpsychires.2016.07.019, PMID: 27491067

[ref40] Kelsey C. M. Prescott S. Mcculloch J. A. Trinchieri G. Valladares T. L. Dreisbach C.. (2021). Gut microbiota composition is associated with newborn functional brain connectivity and behavioral temperament. Brain Behav. Immun. 91, 472–486. doi: 10.1016/j.bbi.2020.11.003, PMID: 33157257

[ref41] Kim H. N. Yun Y. Ryu S. Chang Y. Kwon M. J. Cho J.. (2018). Correlation between gut microbiota and personality in adults: a cross-sectional study. Brain Behav. Immun. 69, 374–385. doi: 10.1016/j.bbi.2017.12.012 29278751

[ref42] Kohn J. N. Snyder-Mackler N. Barreiro L. B. Johnson Z. P. Tung J. Wilson M. E. (2016). Dominance rank causally affects personality and glucocorticoid regulation in female rhesus macaques. Psychoneuroendocrinology 74, 179–188. doi: 10.1016/j.psyneuen.2016.09.005, PMID: 27639059 PMC5494262

[ref43] Kovacic P. Somanathan R. (2013). Sugar toxicity-fundamental molecular mechanisms: α-dicarbonyl, electron transfer, and radicals. J Carbohyd Chem. 32, 105–119. doi: 10.1080/07328303.2012.762102

[ref44] Langille M. G. I. Zaneveld J. Caporaso J. G. Mcdonald D. Knights D. Reyes J. A.. (2013). Predictive functional profiling of microbial communities using 16S rRNA marker gene sequences. Nat. Biotechnol. 31, 814–821. doi: 10.1038/nbt.2676, PMID: 23975157 PMC3819121

[ref45] Lehrke M. Broedl U. C. Biller-Friedmann I. M. Vogeser M. Henschel V. Nassau K.. (2008). Serum concentrations of cortisol, interleukin 6, leptin and adiponectin predict stress induced insulin resistance in acute inflammatory reactions. Crit. Care 12:R157. doi: 10.1186/cc7152, PMID: 19087258 PMC2646322

[ref46] Li Q. Ren Y. Fu X. (2019). Inter-kingdom signaling between gut microbiota and their host. Cell. Mol. Life Sci. 76, 2383–2389. doi: 10.1007/s00018-019-03076-7, PMID: 30911771 PMC11105296

[ref47] Liu C. Cui Y. Li X. Yao M. (2021). *Microeco*: an R package for data mining in microbial community ecology. FEMS Microbiol. Ecol. 97:fiaa255. doi: 10.1093/FEMSEC/FIAA255, PMID: 33332530

[ref48] Liu Z. Dai X. Zhang H. Shi R. Hui Y. Jin X.. (2020). Gut microbiota mediates intermittent-fasting alleviation of diabetes-induced cognitive impairment. Nat. Commun. 11:855. doi: 10.1038/s41467-020-14676-4, PMID: 32071312 PMC7029019

[ref49] Liu X. Li X. Teng T. Jiang Y. Xiang Y. Fan L.. (2022). Comparative analysis of gut microbiota and fecal metabolome features among multiple depressive animal models. J. Affect. Disord. 314, 103–111. doi: 10.1016/J.JAD.2022.06.088, PMID: 35780963

[ref50] Lynch J. B. Hsiao E. Y. (2023). Toward understanding links between the microbiome and neurotransmitters. Ann. N. Y. Acad. Sci. 1524, 10–16. doi: 10.1111/NYAS.14993, PMID: 37017112

[ref51] Magoc T. Salzberg S. L. (2011). FLASH: fast length adjustment of short reads to improve genome assemblies. Bioinformatics 27, 2957–2963. doi: 10.1093/bioinformatics/btr507 21903629 PMC3198573

[ref52] Mallick H. Rahnavard A. Mciver L. J. Ma S. Zhang Y. Nguyen L. H.. (2021). Multivariable association discovery in population-scale meta-omics studies. PLoS Comput. Biol. 17:e1009442. doi: 10.1371/JOURNAL.PCBI.1009442, PMID: 34784344 PMC8714082

[ref53] Millan M. J. (2003). The neurobiology and control of anxious states. Prog. Neurobiol. 70, 83–244. doi: 10.1016/S0301-0082(03)00087-X 12927745

[ref54] Mori H. Maruyama F. Kato H. Toyoda A. Dozono A. Ohtsubo Y.. (2014). Design and experimental application of a novel non-degenerate universal primer set that amplifies prokaryotic 16S rRNA genes with a low possibility to amplify eukaryotic rRNA genes. DNA Res. 21, 217–227. doi: 10.1093/dnares/dst052, PMID: 24277737 PMC3989492

[ref55] Morris G. Berk M. Carvalho A. Caso J. R. Sanz Y. Walder K.. (2017). The role of the microbial metabolites including tryptophan catabolites and short chain fatty acids in the pathophysiology of immune-inflammatory and neuroimmune disease. Mol. Neurobiol. 54, 4432–4451. doi: 10.1007/s12035-016-0004-2, PMID: 27349436

[ref56] Moussawi K. Riegel A. Nair S. Kalivas P. W. (2011). Extracellular glutamate: functional compartments operate in different concentration ranges. Front. Syst. Neurosci. 5:94. doi: 10.3389/fnsys.2011.00094, PMID: 22275885 PMC3254064

[ref57] Nguyen S. T. Minh T. B. Dinh H. T. Le T. D. Nguyen N. P. T. Tran T. T. H.. (2023). Relationship between maternal serum cortisol and maternal insulin resistance and Fetal ultrasound characteristics in gestational diabetes mellitus. Diabetes Metab Syndr Obes. 16, 365–372. doi: 10.2147/dmso.S400995, PMID: 36788989 PMC9922503

[ref58] Ortega V. A. Mercer E. M. Giesbrecht G. F. Arrieta M.-C. (2021). Evolutionary significance of the neuroendocrine stress Axis on vertebrate immunity and the influence of the microbiome on early-life stress regulation and health outcomes. Front. Microbiol. 12:634539. doi: 10.3389/fmicb.2021.634539 33897639 PMC8058197

[ref59] Park E. Yun K. E. Kim M. H. Kim J. Chang Y. Ryu S.. (2021). Correlation between gut microbiota and six facets of neuroticism in Korean adults. J. Pers. Med. 11:1246. doi: 10.3390/JPM11121246, PMID: 34945718 PMC8704006

[ref60] Partrick K. A. Chassaing B. Beach L. Q. Mccann K. E. Gewirtz A. T. Huhman K. L. (2018). Acute and repeated exposure to social stress reduces gut microbiota diversity in Syrian hamsters. Behav. Brain Res. 348:277. doi: 10.1016/j.bbr.2018.03.044, PMID: 29474810 PMC6246037

[ref61] Patel C. Sugimoto K. Douard V. Shah A. Inui H. Yamanouchi T.. (2015). Effect of dietary fructose on portal and systemic serum fructose levels in rats and in KHK^−/−^ and GLUT5^−/−^ mice. Am. J. Physiol. Gastrointest. Liver Physiol. 309, G779–G790. doi: 10.1152/ajpgi.00188.2015, PMID: 26316589 PMC4628968

[ref62] Peerboom C. Wierenga C. J. (2021). The postnatal GABA shift: a developmental perspective. Neurosci. Biobehav. Rev. 124, 179–192. doi: 10.1016/J.NEUBIOREV.2021.01.024, PMID: 33549742

[ref63] Pritchard A. J. Sheeran L. K. Gabriel K. I. Li J.-H. Wagner R. S. (2014). Behaviors that predict personality components in adult free-ranging Tibetan macaques *Macaca thibetana*. Curr Zool. 60, 362–372. doi: 10.1093/czoolo/60.3.362

[ref64] Rangassamy M. Dalmas M. Feron C. Gouat P. Roedel H. G. (2015). Similarity of personalities speeds up reproduction in pairs of a monogamous rodent. Anim. Behav. 103, 7–15. doi: 10.1016/j.anbehav.2015.02.007

[ref65] Rawlings B. Flynn E. Freeman H. Reamer L. Schapiro S. J. Lambeth S.. (2020). Sex differences in longitudinal personality stability in chimpanzees. Evol Human Sci. 2:e46. doi: 10.1017/EHS.2020.45, PMID: 37588391 PMC10427468

[ref66] Revelle W. (2023). Psych: procedures for psychological, psychometric, and personality research. Northwestern University. R package version 2.3.9. Available at: https://CRAN.R-project.org/package=psych

[ref67] Ruhe H. G. Mason N. S. Schene A. H. (2007). Mood is indirectly related to serotonin, norepinephrine and dopamine levels in humans: a meta-analysis of monoamine depletion studies. Mol. Psychiatry 12, 331–359. doi: 10.1038/SJ.MP.4001949, PMID: 17389902

[ref68] Savignac H. M. Kiely B. Dinan T. G. Cryan J. F. (2014). *Bifidobacteria* exert strain-specific effects on stress-related behavior and physiology in BALB/c mice. Neurogastroenterol. Motil. 26, 1615–1627. doi: 10.1111/nmo.12427, PMID: 25251188

[ref69] Sgritta M. Dooling S. W. Buffington S. A. Momin E. N. Francis M. B. Britton R. A.. (2019). Mechanisms underlying microbial-mediated changes in social behavior in mouse models of autism Spectrum disorder. Neuron 101, 246–259.e6. doi: 10.1016/j.neuron.2018.11.018, PMID: 30522820 PMC6645363

[ref70] Sharon G. Sampson T. R. Geschwind D. H. Mazmanian S. K. (2016). The central nervous system and the gut microbiome. Cell 167, 915–932. doi: 10.1016/j.cell.2016.10.027, PMID: 27814521 PMC5127403

[ref71] Silva Y. P. Bernardi A. Frozza R. L. (2020). The role of short-chain fatty acids from gut microbiota in gut-brain communication. Front. Endocrinol. 11:25. doi: 10.3389/fendo.2020.00025, PMID: 32082260 PMC7005631

[ref72] Simpson C. A. Diaz-Arteche C. Eliby D. Schwartz O. S. Simmons J. G. Cowan C. S. M. (2021). The gut microbiota in anxiety and depression - a systematic review. Clin. Psychol. Rev. 83:101943. doi: 10.1016/j.cpr.2020.101943 33271426

[ref73] Slipogor V. Massen J. J. M. Schiel N. Souto A. Bugnyar T. (2021). Temporal consistency and ecological validity of personality structure in common marmosets (*Callithrix jacchus*): a unifying field and laboratory approach. Am. J. Primatol. 83:e23229. doi: 10.1002/ajp.23229, PMID: 33464603 PMC7900989

[ref74] Stilling R. M. Van De Wouw M. Clarke G. Stanton C. Dinan T. G. Cryan J. F. (2016). The neuropharmacology of butyrate: the bread and butter of the microbiota-gut-brain axis? Neurochem. Int. 99, 110–132. doi: 10.1016/j.neuint.2016.06.011, PMID: 27346602

[ref75] Sueur C. Petit O. (2008). Shared or unshared consensus decision in macaques? Behav. Process. 78, 84–92. doi: 10.1016/j.beproc.2008.01.004 18281161

[ref76] Suomi S. J. Novak M. A. Well A. (1996). Aging in rhesus monkeys: different windows on behavioral continuity and change. Dev. Psychol. 32, 1116–1128. doi: 10.1037/0012-1649.32.6.1116

[ref77] Taylor A. M. Thompson S. V. Edwards C. G. Musaad S. M. A. Khan N. A. Holscher H. D. (2020). Associations among diet, the gastrointestinal microbiota, and negative emotional states in adults. Nutr. Neurosci. 23, 983–992. doi: 10.1080/1028415X.2019.1582578, PMID: 30794085

[ref78] Valles-Colomer M. Falony G. Darzi Y. Tigchelaar E. F. Wang J. Tito R. Y.. (2019). The neuroactive potential of the human gut microbiota in quality of life and depression. Nat. Microbiol. 4, 623–632. doi: 10.1038/s41564-018-0337-x, PMID: 30718848

[ref79] Walsh J. J. Christoffel D. J. Heifets B. D. Ben-Dor G. A. Selimbeyoglu A. Hung L. W.. (2018). 5-HT release in nucleus accumbens rescues social deficits in mouse autism model. Nature 560, 589–594. doi: 10.1038/s41586-018-0416-4, PMID: 30089910 PMC8164568

[ref80] Wang Y. Li N. Yang J.-J. Zhao D.-M. Chen B. Zhang G.-Q.. (2020). Probiotics and fructo-oligosaccharide intervention modulate the microbiota-gut brain axis to improve autism spectrum reducing also the hyper-serotonergic state and the dopamine metabolism disorder. Pharmacol. Res. 157:104784. doi: 10.1016/j.phrs.2020.104784, PMID: 32305492

[ref81] Wei W. Lu J. G. Galinsky A. D. Wu H. Gosling S. D. Rentfrow P. J.. (2017). Regional ambient temperature is associated with human personality. Nat. Hum. Behav. 1, 890–895. doi: 10.1038/s41562-017-0240-0 31024181

[ref82] Weiss A. Staes N. Pereboom J. J. M. Inoue-Murayama M. Stevens J. M. G. Eens M. (2015). Personality in bonobos. Psychol. Sci. 26, 1430–1439. doi: 10.1177/0956797615589933 26209530

[ref83] Wheeler P. R. Brosch R. Coldham N. G. Inwald J. K. Hewinson R. G. Gordon S. V. (2008). Functional analysis of a clonal deletion in an epidemic strain of *Mycobacterium bovis* reveals a role in lipid metabolism. Microbiol-sgm 154, 3731–3742. doi: 10.1099/mic.0.2008/022269-0 19047741

[ref84] Wolf M. Weissing F. J. (2012). Animal personalities: consequences for ecology and evolution. Trends Ecol. Evol. 27, 452–461. doi: 10.1016/j.tree.2012.05.001 22727728

[ref85] Wu M. Chen S. Sun B. Wang X. Xia D. Li J.-H. (2021). Effects of seasonality and social ranks on fecal cortisol levels in male Tibetan macaques (*Macaca thibetana*). Acta Theriol Sin. 41, 398–405. doi: 10.16829/j.slxb.150524

[ref86] Yang J. Fu X. Liao X. Li Y. (2020). Effects of gut microbial-based treatments on gut microbiota, behavioral symptoms, and gastrointestinal symptoms in children with autism spectrum disorder: a systematic review. Psychiatry Res. 293:113471. doi: 10.1016/j.psychres.2020.113471, PMID: 33198044

[ref87] Zhang Q.-X. Li J.-H. Xia D.-P. Zhu Y. Wang X. Zhang D. (2014). Influence of dominance rank and affiliation relationships on self-directed behavior in female Tibetan macaques (*Macaca thibetana*). Zool. Res. 35, 214–221. doi: 10.11813/j.issn.0254-5853.2014.3.214, PMID: 24866492 PMC5055544

