# Supplementary material for: Gut microbiome is associated with personality traits of free-ranging Tibetan macaques (Macaca thibetana)
Source: Front Microbiol. 2024 Apr 22;15:1381372. doi: 10.3389/fmicb.2024.1381372 (PMC11070476; doi:10.3389/fmicb.2024.1381372)
Supplement: Supplementary file 1 [file Data_Sheet_1.PDF]

Supplementary Material

1 Supplementary Figures and Tables

1.1 Supplementary Figures

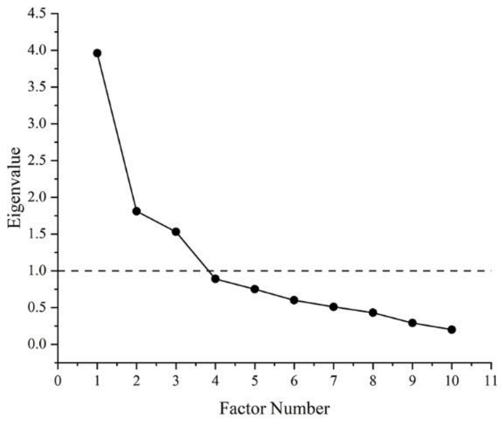

Supplementary Figure 1. Scree plots generated by principal component analysis of 24 Tibetan macaques Above the dashed line, three factors with eigenvalues greater than 1 are selected

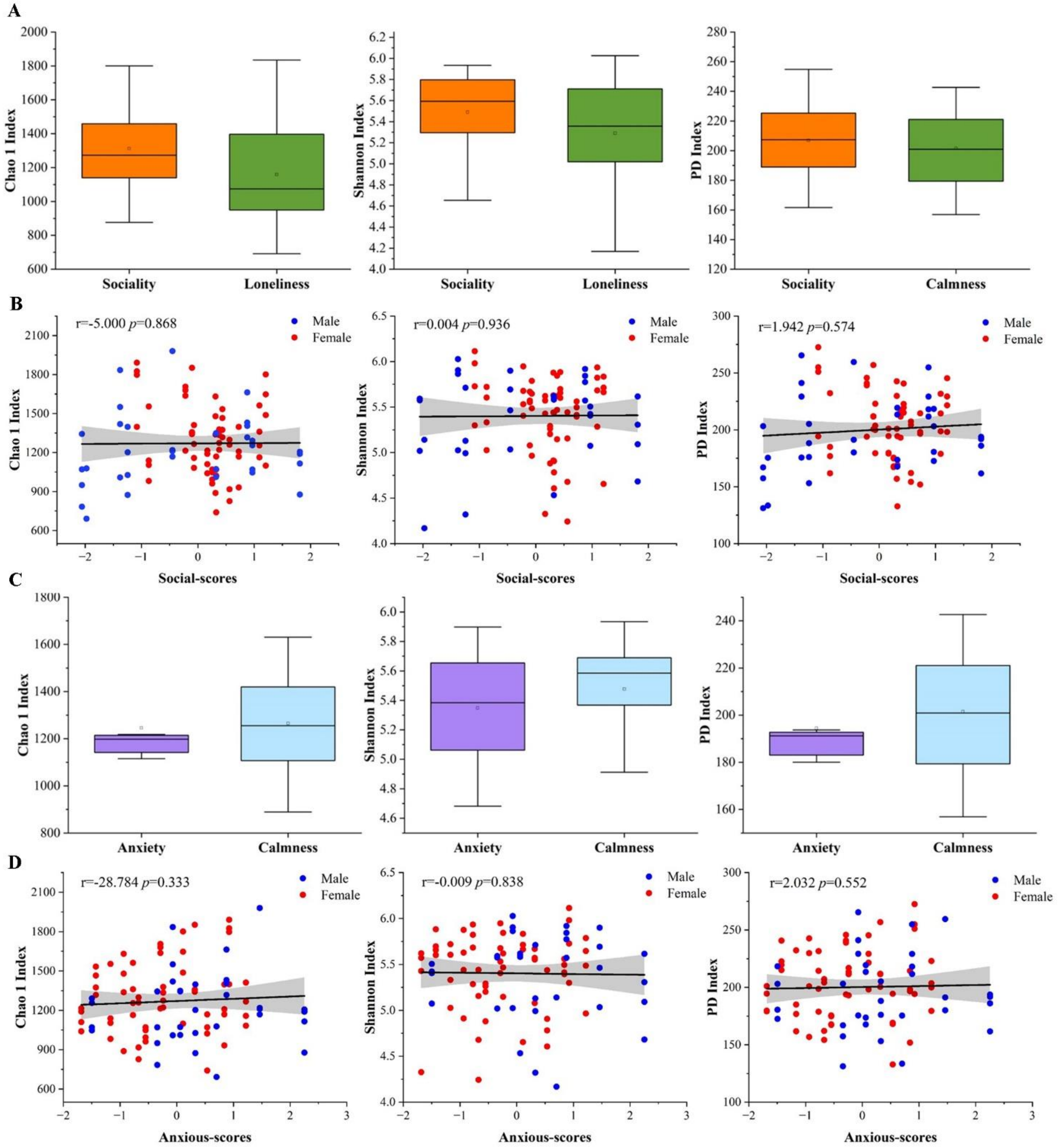

Supplementary Figure 2. Comparison of alpha diversity in different personality dimensions (Social and Anxious). Chao 1 index, Shannon index, and PD index were used for alpha diversity. A&C: Qualitative analysis of alpha diversity in Social and Anxious dimension, C&D: Quantitative analysis of alpha diversity in Social and Anxious dimension. Shaded areas represent 95% confidence intervals

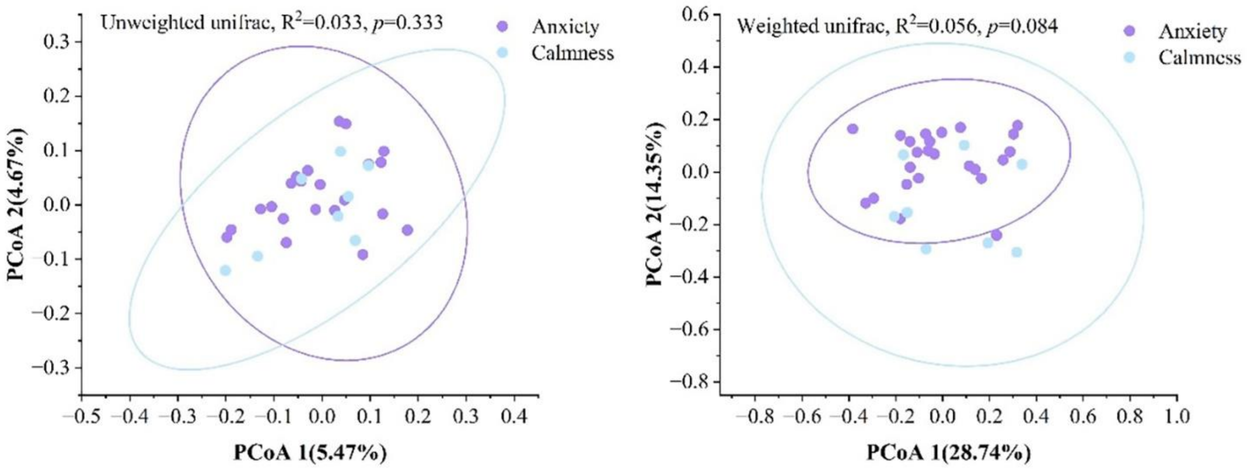

Supplementary Figure 3. Comparison of the β diversity between Anxiety and Calmness based on weighted and unweighted distances. A: based on unweighted UniFrac distance, B: based on weighted UniFrac distance. The ellipses represent a 95% confidence interval for each group.

1.2 Supplementary Tables

Supplementary Table 1. Population information of adult Tibetan macaques group YA1

Male			Female		
Name	Immigration/Native	Age	Name	Immigration/Native	Age
BHZ	Immigration	14	TFH	Native	7
DB	Immigration	8	TH	Native	19
DZ	Immigration	8	THX	Native	10
NM	Immigration	12	THY	Native	14
TQ	Immigration	11	TQG	Native	6
TQS	Native	8	TQL	Native	10
WM	Immigration	15	TQY	Native	7
YL	Immigration	8	TXH	Native	12
YXK	Native	10	TXX	Native	15
			YCH	Immigration	10
			YCL	Native	10
			YCY	Native	14
			YH	Native	19
			YXX	Native	13
			YXY	Native	8

Immigration: Individuals migrating from other groups into this group
Native: Individuals that have been growing within this group all along

Supplementary Table 2. Behavioral definitions

Behavior	Defined
Approach	An individual moves directly toward another individual, coming within 1 meter. It's a friendly act.
Avoid	The animal twists its body to the individual who attacked it and poses to escape. At the same time, its mouth is open and the corners of its mouth are elongated down, showing a frightened appearance. Avoidance is an act of submission
Bite	The performer grabs the receiver tightly, preventing him/her from fleeing, and bites the recipient vigorously. This is the most violent attack.
Bridge	Bridging involves three individuals, an infant or young juvenile, and a) two adult males, b) one adult male and one subadult male, or c) two adult females. The two older individuals hold the infant on its back, lower their heads, and lick the belly and/or genitals of the infant. They will often teeth-chatter and vocalize excitedly. The infant is usually male and his penis becomes erect immediately. Other infants, particularly females will approach a bridging triad with excitement as if they wish to participate.
Chase	An individual stares at the recipient and rushes at him/her at great speed. The recipient typically flees. Chasing is an act of aggression.
Flee	The target of an attack will run in the opposite direction from the attacker.
Groom	An individual uses his/her fingers and palms to groom the fur of another individual. The groomer may pick out small objects from the recipient's fur and eat them.
Ground slap	An individual supports with one hand on the ground, the other flaps the ground, and stares at the other male individual, then it looks down.
Leave	An individual moves from within 1 meter to more than 1 meter of another individual.
Present	One individual approach the front of the other and shows his bottom to the other. Usually, the presenter is the lower-ranking of the two partners. Females also present to males.
Proximity	Two or more individuals are sitting or lying within 1 meter of one another.
Redirection	When A is attacked by B, A responds immediately by attacking C. C is an individual that is nearby and lower-ranking than both A and B. In some cases, B will join the attack against C. The behavior is an advanced behavioral strategy.
Seizing	One individual grabs the hair of the body, face, or neck of another one, or it may simply grab its ears. Shake it back and forth a few times before letting go. Sometimes the actors hold their heads close to each other while grasping each other, and stare at each other closely. This is a relatively fierce attack.
Self-groom	Picking through and slowly brushing aside fur with one or both hands.
Self-scratch	Movement of the hand or foot during which the fingertips are drawn across the fur or skin.
Sit alone	An individual sits alone with no other individuals around.
Stare	An individual looks directly at another individual with its eyes wide open and with its shoulders raised for about 3-5 seconds. The staring individual appears as if it is preparing to lunge or chase the recipient of the stare. This behavior is when an individual makes a mild attack on other individuals.

Supplementary Table 3. Factor scores of adult individuals in YA1 group in different personality dimensions

Individual	Social	Shy	Anxious
BHZ	-1.975	-1.097	0.703
DB	-2.058	-1.356	-0.341
DZ	-1.379	0.057	-0.070
NM	0.876	-0.105	0.879
TFH	-0.871	0.293	-1.168
TH	0.256	-0.421	-0.548
THX	-1.081	0.805	0.924
THY	-0.103	1.431	0.319
TQ	0.324	-0.389	0.062
TQG	1.093	0.065	-0.771
TQL	0.326	0.450	0.537
TQS	-0.450	0.975	1.463
TQY	-0.220	0.503	-0.287
TXH	0.381	0.590	-0.239
TXX	0.313	1.217	-0.936
WM	-1.245	-1.318	0.328
YCH	0.727	0.649	0.840
YCL	-0.069	1.010	1.219
YCY	0.563	-0.337	-0.672
YH	0.168	0.653	-1.685
YL	0.970	-2.487	-1.495
YXK	1.811	-1.681	2.253
YXX	1.206	-0.363	0.111
YXY	0.436	0.855	-1.426

Supplementary Table 4. Correlation between personality dimensions and alpha diversity index

Personality dimension	N	Chao 1		Shannon		PD	
		β	*p*-value	β	*p*-value	β	*p*-value
Social	94	-5.000	0.868	0.004	0.936	1.942	0.574
Shy	94	116.707	0.001***	0.146	0.009**	12.888	0.002**
Anxious	94	28.784	0.333	-0.009	0.838	2.032	0.552

N: the number of samples, β: Regression coefficients
** *p*-value <= 0.01, *** *p*-value <= 0.001
test: General Linear Model

Supplementary Table 5. Differences in the alpha diversity index between personality groups

Personality Group	N	Chao 1	Shannon	PD
Sociality & Loneliness	20&14	p = 0.116	p = 0.359	p = 0.064
Shyness & Boldness	40&8	p = 0.004**	p = 0.016*	p = 0.027*
Anxiety & Calmness	8&24	p = 0.840	p = 0.400	p = 0.509

N: the number of samples, * p-value <= 0.05, ** p-value <= 0.01

Supplementary Table 6. Correlations of microbial taxa with personality traits as a quantitative (continuous) variable using MaAsLin2 analysis

Personality	Family	Genus	CE	SD	*p*-value	*q*-value	N/N not zero
Social	Akkermansiaceae		0.701	0.236	3.80E-03	0.069	94/20
	Akkermansiaceae	*Akkermansia*	0.701	0.236	3.80E-03	0.069	94/20
	Desulfovibrionaceae		0.666	0.184	4.80E-04	0.031	94/80
	Enterobacteriaceae		-0.811	0.272	3.70E-03	0.069	94/61
	Enterobacteriaceae	*Escherichia.Shigella*	-0.798	0.272	4.23E-03	0.072	94/58
	Erysipelatoclostridiaceae	*Asteroleplasma*	0.496	0.170	4.41E-03	0.073	94/94
	Prevotellaceae	*Prevotellaceae_UCG.003*	0.483	0.168	5.10E-03	0.079	94/94
	Succinivibrionaceae		0.611	0.217	5.95E-03	0.089	94/94
	Succinivibrionaceae	*Succinivibrio*	0.611	0.218	6.18E-03	0.090	94/94
	Veillonellaceae		0.701	0.162	4.00E-05	0.010	94/93
	Veillonellaceae	*Dialister*	0.640	0.166	2.20E-04	0.022	94/93
Shy	Oscillospiraceae		0.222	0.067	1.31E-03	0.073	94/94

CE: Coefficient, N: the number of samples, SD: Standard Deviation

The results with *q* -value <0.1 were listed.

The *q* -values were calculated using Benjamini-Hochberg correction (FDR).

Supplementary Table 7. Differences of microbial taxa with personality traits as a qualitative (binary) variable using MaAsLin2 analysis

Personality Group	Family	Genus	N	U	*p*-value
Sociality & Loneliness	Akkermansiaceae		20&14	110	0.207
	Akkermansiaceae	*Akkermansia*	20&14	110	0.207
	Desulfovibrionaceae		20&14	60.5	0.005
	Enterobacteriaceae		20&14	72.5	0.009
	Enterobacteriaceae	*Escherichia.Shigella*	20&14	70	0.006
	Erysipelatoclostridiaceae	*Asteroleplasma*	20&14	63.5	0.007
	Prevotellaceae	*Prevotellaceae_UCG.003*	20&14	80	0.036
	Succinivibrionaceae		20&14	77	0.027
	Succinivibrionaceae	*Succinivibrio*	20&14	76	0.025
	Veillonellaceae		20&14	45	0.001
	Veillonellaceae	*Dialister*	20&14	57	0.004
Shyness & Boldness	Oscillospiraceae		40&8	86	0.04

N: the number of samples, U: U-statistic
test: Mann-Whitney' U
